# Social support and cognitive function in Chinese older adults who experienced depressive symptoms: is there an age difference?

**DOI:** 10.3389/fnagi.2023.1175252

**Published:** 2023-05-12

**Authors:** Yurong Jing, Wei Wang, Wenjia Peng, Meng Wang, Xiaoli Chen, Xinya Liu, Pengfei Wang, Fei Yan, Yinghua Yang, Xinguo Wang, Shuangyuan Sun, Ye Ruan, Ying Wang

**Affiliations:** ^1^School of Public Health, Fudan University, Shanghai, China; ^2^Key Laboratory of Health Technology Assessment, National Health and Family Planning, Commission of the People’s Republic of China, Fudan University, Shanghai, China; ^3^Shanghai Center for Clinical Laboratory, Shanghai, China; ^4^College of Public Health, Shanghai University of Medicine and Health Sciences, Shanghai, China; ^5^Shanghai Municipal Center for Disease Control and Prevention, Shanghai, China

**Keywords:** cognitive function, depressive symptoms, social support, age difference, objective support, subjective support, support utilization

## Abstract

**Objective:**

This study examined the moderating effect of overall social support and the different types of social support on cognitive functioning in depressed older adults. We also investigated whether the moderating effect varied according to age.

**Methods:**

A total of 2,500 older adults (≥60 years old) from Shanghai, China were enrolled using a multistage cluster sampling method. Weighted linear regression and multiple linear regression was utilized to analyze the moderating effect of social support on the relationship between depressive symptoms and cognitive function and to explore its differences in those aged 60–69, 70–79, and 80 years and above.

**Results:**

After adjusting for covariates, the results indicated that overall social support (β = 0.091, *p* = 0.043) and support utilization (β = 0.213, *p* < 0.001) moderated the relationship between depressive symptoms and cognitive function. Support utilization reduced the possibility of the cognitive decline in depressed older adults aged 60–69 years (β = 0.310, *p*  < 0.001) and 80 years and above (β = 0.199, *p*  < 0.001), while objective support increased the possibility of cognitive decline in depressed older people aged 70–79 years (β = −0.189, *p*  < 0.001).

**Conclusion:**

Our findings highlight the buffering effects of support utilization on cognitive decline in depressed older adults. We suggest that age-specific measures should be taken when providing social support to depressed older adults in order to reduce the deterioration of cognitive function.

## 1. Introduction

The incidence of dementia is increasing at a rapid rate, with approximately 51.6 million people living with Alzheimer’s disease and related dementias (ADRD) worldwide in 2019, and China accounting for more than 1/4 (Ren et al., 2022). Although the relationship between depressive symptoms and cognitive decline may be bi-directional, a growing body of studies have indicated that depressive symptoms are a prodrome of dementia and play a significant role in the development of long-term cognitive impairment (Freire et al., 2017; Livingston et al., 2020; Oh et al., 2021). Therefore, we believe that it is imperative to explore ways to moderate the relationship between depressive symptoms and cognitive function.

In the framework of active aging proposed by the World Health Organization, improving social support is regarded as a vital factor in enhancing health, independence, and productivity during the aging process (World Health Organization, 2002). Previous studies have shown that social support influences cognitive function and depressive symptoms in older adults, with higher levels of social support being associated with significantly higher levels of cognitive function (i.e., global cognitive function, episodic memory, working memory, and executive function) (Salinas et al., 2017; Shaoqing et al., 2017). The buffering model of social support argues that social support can play a protective role in mental health by lessening the negative psychological consequences of stressful events (Peggy, 2011). A prospective cohort study in Japan found that a lack of social support was significantly associated with an increased risk of depression (Koizumi, 2005). Whether social support can moderate the relationship between depressive symptoms and cognition function is still unclear.

Previous studies have shown that different types of social support have different effects on cognition and depression (Ellwardt et al., 2013; Jin et al., 2020). Social support can be divided into objective, subjective and support utilization by nature (Xiao, 1994). Studies have shown that higher levels of emotional support may be associated with better verbal memory and global cognition through larger hippocampal volume (HPV) and that emotional social support’s protective effect on cognitive decline is greater than that of instrumental support (Ellwardt et al., 2013; Kim et al., 2019). In addition, research indicates that perception of support is more significant to an individual than the amount of objective support received in predicting emotional well-being (Jang et al., 2002). Support utilization can help promote the resolution of individual problems through the use of behavioral problem-oriented coping methods, which is conducive to the improvement of depressive symptoms in older adults (Thoits, 1986; Jin et al., 2020). Therefore, it is more essential to understand how different types of social support function when investigating the moderating effects of social support on the relationship between depressive symptoms and cognitive functioning to adjust support strategies in a targeted manner.

Social support may have different effects on older adults’ health due to the different age groups they are in (Litwin and Stoeckel, 2013; Qiang and Liu, 2022). A study has shown that objective and subjective support have significant positive effects on the mental health of older adults aged 70–79 years and ≥ 80 years, while support utilization has greater effects on the health of older adults aged 60–69 years (Qiang and Liu, 2022). In respect of older adults over the age of 80 years, it has been found that living with a spouse actually reduced their quality of life, but the opposite was true for those aged 60–79 years who experienced an improvement in their quality of life (Litwin and Stoeckel, 2013). Given these diverse findings, we suggest that the moderating effect of social support on the relationship between depressive symptoms and cognition may vary across the different age groups.

**Figure 1:**
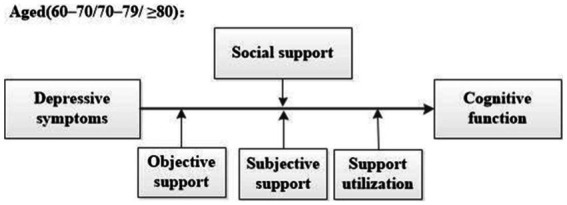
Hypothesized model.

The aims of this study are to explore the moderating role of overall social support on the relationship between depressive symptoms and cognitive function as well as how the different types of social support moderating this relationship. In addition, we aim to investigate how overall and different types of social support play a moderating role in the relationship between depressive symptoms and cognitive function in older adults of different age groups. We propose the following specific hypotheses (Figure 1):

*Hypothesis 1*: The relationship between depressive symptoms and cognitive function can be moderated by social support, overall and by the different types (objective support, subjective support, and support utilization) of social support, and the effects will vary.

*Hypothesis 2*: The moderating effects in Hypothesis 1 will vary by age.

## 2. Materials and methods

### 2.1. Data source and participants

This study was conducted in Jiangwan Town, Hongkou District, Shanghai during the period December 2020 to July 2021. A total of 2,500 participants aged 60 years and older took part in our study. The seventh national census of China showed that Shanghai has the second highest proportion of older adults aged 60 years and above in the country, calculated to be 23.38% of the population (Statistics, 2020). This study adopted a multi-stage cluster random sampling method, and older adults were stratified by gender and age. We selected one of the 35 neighborhoods in Jiangwan at random. If the population did not meet the needs of the sample, the next neighborhood would be randomly selected until the sample number met the requirements. Data were collected face-to-face by trained interviewers, using a structured questionnaire.

Our inclusion criteria were as follows: participants needed to be (1) aged ≥60 years; (2) permanent residents who lived in Jiangwan Town, Hongkou District for more than 6 months in the past year; (3) conscious, articulate, and able to communicate normally with investigators; and (4) able to give informed consent. Our exclusion criterion were as follows: (1) older adults who did not live in the community or were separated from their registered permanent residence during the research period; (2) older adults who are blind, deaf, or severely hearing impaired; (3) older adults who have serious diseases that could affect brain function or cognitive function evaluation; (4) older adults with severe perceptual impairment who are unable to complete cognitive function assessments; and (5) older adults who are at the end of life or have other circumstances that prevent them from completing the questionnaire.

The protocol for this study was approved by the Ethical Review Board of Fudan University (NO. IRB#TYSQ 2019-2-03), and all participants provided informed consent.

### 2.2. Measures

#### 2.2.1. Cognitive function

Cognitive function was assessed using the Mini-Mental State Examination (MMSE), which is the most commonly used tool to screen for cognitive impairment and dementia worldwide and is suitable for the older population (Folstein et al., 1975; Shulman et al., 2006; McCann et al., 2018; Lv et al., 2019). This scale evaluates the following seven dimensions: orientation to time, orientation to place, immediate memory, delayed memory, attention and computation, language, and spatial vision. It contains 30 items and each item is scored either 0 or 1. The total score for the MMSE ranges from 0 to 30, with a higher score indicating better cognitive functioning. In this study, Cronbach’s coefficient of the MMSE was 0.842.

#### 2.2.2. Depressive symptoms

Depressive symptoms were measured utilizing the Geriatric Depression Scale (GDS-30) (Yesavage et al., 1982). It contains 30 items and each item is scored either 0 or 1. The total score for the GDS-30 ranges from 0 to 30, with a higher score indicating greater depressive symptoms. The cut-off score for depressive symptoms was 11 (Yesavage et al., 1982). The Chinese version of the GDS-30 has good reliability and validity for Chinese urban community-dwelling older adults (Jie et al., 2013). In this study, Cronbach’s coefficient of the GDS-30 was 0.921.

#### 2.2.3. Social support

Social support was assessed using the Social Support Rating Scale (SSRS). It contains 10 items divided into 3 dimensions: objective support, subjective support, and support utilization. For items 1 to 4 and 8 to 10, each item is scored from 1 to 4. Item 5 is divided into 5 options, each option is scored from 1 to 4. In respect of items 6 and 7, each item is scored from 0 to 9. The scores of total, objective support, subjective support, and support utilization range from 12 to 66, 1 to 22, 8 to 32, and 3 to 12, respectively. A higher score indicates a greater level of social support. Previous studies have shown that the SSRS has highly reliability and validity and is widely used in the Chinese population (Qin et al., 2021; Tao et al., 2022). In this study, Cronbach’s coefficient of the SSRS was 0.692.

#### 2.2.4. Covariates

Cognitive function-associated sociodemographic variables, health behaviors, and health status variables were considered as covariates in our analysis, which have previously been reported to be associated with cognitive function among older adults (Durazzo et al., 2014; Koch et al., 2019; Lutsey et al., 2019; Zhang et al., 2020; Becker et al., 2022; Li et al., 2022; Nerobkova et al., 2022; Yu et al., 2022; Zhang et al., 2022; Bloomberg et al., 2023; Du et al., 2023). Sociodemographic variables included gender (male or female), age (60–69 years, 70–79 years, or ≥ 80 years), education level (illiterate/primary school, junior school, senior high school, or college or above), marital status (unmarried/divorced/widowed or married), living arrangement (alone or with others), household income (≤ 5,000, 5,001 – 10,000, or > 10,000). Health behavior variables included smoking status (never/past or current) and alcohol drinking status (never/past or current). Health status variables included chronic conditions (no/yes) and activities of daily living (normal/impaired). The Physical Self-Maintenance Scale (PSMS) and Instrumental Activities of Daily Living Scale (IADL) were used to assess the activities of daily living (ADL) and IADL, respectively (Lawton and Brody, 1969). PSMS contains 6 items and IADL contains 8 items. Impairment of any item of the PSMS/IADL is defined as impairment of the ADL/IADL (Yang et al., 2014). In China, older people aged 60 and over can be categorized into young-old (60–69 years), middle-old (70–79 years) and oldest-old (≥80 years), and they differ in living arrangement, physical functions, health status (including psychology) and other aspects (Choi et al., 2017; Aimo et al., 2020; Vélez et al., 2020; Tao et al., 2023).

### 2.3. Statistical analysis

Statistical analyses were performed by using Stata, version 14.0 (StataCorp LLC, College Station, TX) and all the analyses were set at 0.05 significance level. First, descriptive analyses included frequency and percentage for categorical variables and mean and standard deviation for continuous variables. Second, spearman correlation analysis was used to explore the associations between depressive symptoms, social support (total social support, objective support, subjective support, and support utilization), covariates and cognitive function, correlation matrixes are in Supplementary Tables S1–S4. Third, weighted linear regression analysis was used to test whether social support (total social support, objective support, subjective support, and support utilization) moderated the association between depressive symptoms and cognitive function. Depressive symptoms and covariates were added in model 1; social support and it’s three types were added in model 2 and 4, respectively; and interaction term of depressive symptoms and social support and its three types were added in model 3 and 5, respectively. Third, moderating effects of social support and it’s three types on the association between depressive symptoms and cognitive function under different age groups (60–69 years, 70–79 years, and ≥ 80 years) were examined with multiple linear regression analysis. The specific steps were the same as the second step described above. Variance inflation factors of all models range from 1.45 to 3.21, indicating acceptable levels of multicollinearity (Peat and Barton, 2005).

## 3. Results

Table 1 shows the descriptive characteristics of the participants. Of the 2,500 participants, 51.6% were female, and most of the participants were aged 60–69 years (58.9%), had a senior high school level of education (39.6%), were married (85.6%), were living with others (94.0%), and had a household income of 5,001–10,000 (57.4%). In addition, more than half of the participants were non-smokers (85.6%) and 89.0% of the participants did not drink alcohol. Over half of the participants did not have an activity disorder (normal ADL: 56.2%, normal IADL: 72.6%), and 89.0% of the participants did not have depressive symptoms. The mean score of total social support (subjective support, objective support, and support utilization) was 39.53 ± 6.32 (8.85 ± 2.53, 23.48 ± 4.27, 7.2 ± 2.2 2, respectively), and the mean score of cognitive function was 28.44 ± 4.29.

**Table 1:** Sample characteristics.

Variables	N/M	%/SD	Variables	N/M	%/SD
**Total**	2,500	100.0	**Smoking status**		
**Gender**			Never/Past	2,139	85.6
Male	1,209	48.4	Current	361	14.4
Female	1,291	51.6	**Alcohol drinking status**		
**Age**			Never/Past	2,226	89.0
60–69	1,473	58.9	Current	274	11.0
70–79	670	26.8	**Chronic conditions**		
≥ 80	357	14.3	No	1,010	40.4
**Education**			Yes	1,490	59.6
Illiterate / Primary school	268	10.7	**ADL**		
Junior school	909	36.4	Normal	1,405	56.2
Senior high school	990	39.6	Impaired	1,095	43.8
College or above	333	13.3	**IADL**		
**Marital status**			Normal	1816	72.6
Unmarried/divorced/widowed	359	14.4	Impaired	684	27.4
Married	2,141	85.6	**Depressive symptoms**		
**Living arrangement**			No	2,224	89.0
Alone	150	6.0	Yes	276	11.0
With others	2,350	94.0	**Social support**	39.53	6.32
**Household income**			Objective support	8.85	2.53
≤ 5,000	304	12.2	Subjective support	23.48	4.27
5,001-10,000	1,434	57.4	Support utilization	7.2	2.22
> 10,000	762	30.5	**Cognitive function**	28.44	4.29

ADL, Activities of daily living; IADL, Instrumental activities of daily living; SD, Standard deviation.

**Figure 2:**
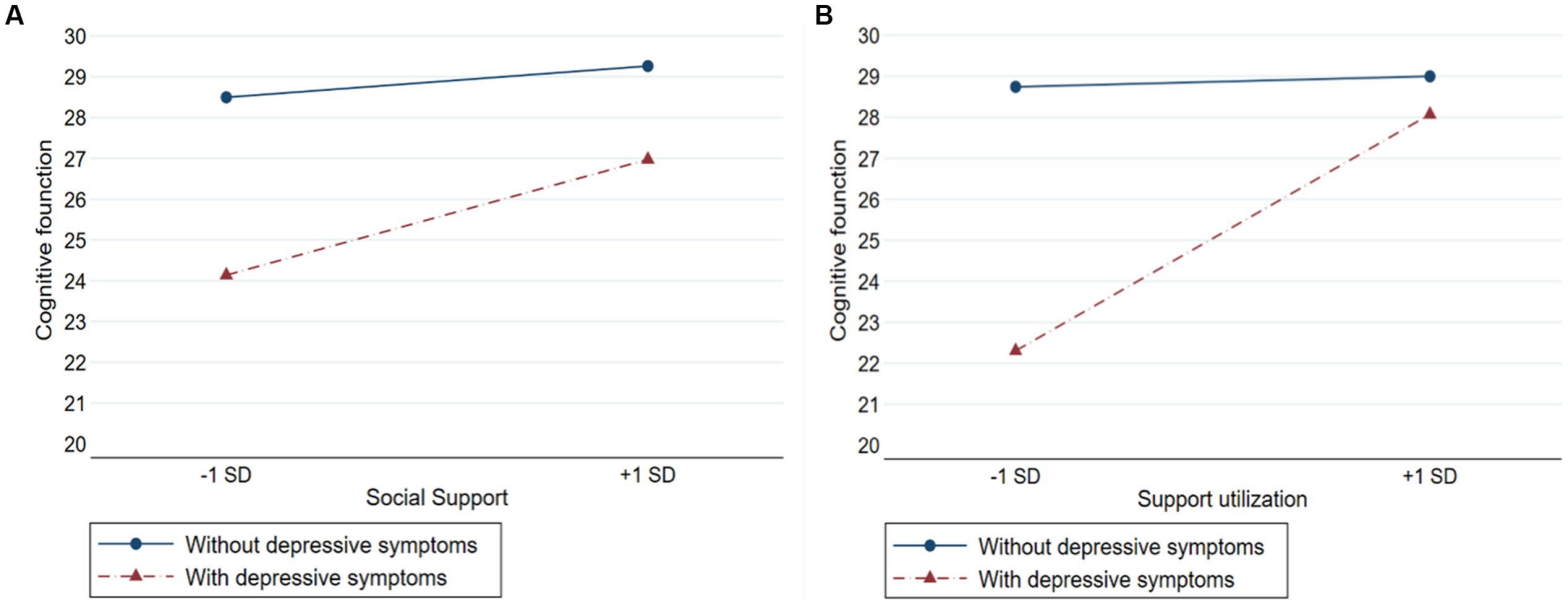
Moderation of social support to depressive symptoms and cognitive function. **(A)** Represents depressive symptoms × social support. **(B)** Represents depressive symptoms × support utilization.

Table 2 shows the moderating effect of social support on the relationship between depressive symptoms and cognitive function. First, model 1 included depressive symptoms and covariates. The results showed that depressive symptoms were significantly negatively associated to cognitive function, older people with depressive symptoms had lower levels of cognitive function than those without depressive symptoms (*β* = −0.286, *p* < 0.001). Model 2 further included social support and showed it was significantly positively associated with cognitive function (β = 0.108, p < 0.001). Model 3 included the interaction term (depressive symptoms × social support) to explore whether social support played a moderating role in the relationship between depressive symptoms and cognitive function. The significant interaction term (*β* = 0.091, *p* = 0.043) indicated that social support reduced the possibility of cognitive decline in older adults with depressive symptoms. Figure 2A plots the estimated effects of social support on cognitive function by depressive symptoms. In the absence of social support, the cognitive score of older adults with depressive symptoms was 24.14, and that of without depressive symptoms older adults was 28.49. When social support was at the M + 1SD level, the cognitive score of older adults with depressive symptoms was 26.97, and that of without depressive symptoms older adults was 29.26.

**Table 2:** Moderating effects of social support (objective support, subjective support, and support utilization) on the relationship between depressive symptoms and cognitive function.

	Model 1		Model 2		Model 3		Model 4		Model 5	
	*β*	*p*-value	*β*	*p*-value	*β*	*p*-value	*β*	*p*-value	*β*	*p*-value
**Depressive symptoms (ref: no)**																				
Yes	−0.286	<0.001	−0.277	<0.001	−0.241	<0.001	−0.279	<0.001	−0.252	<0.001
**Social support**			0.108	<0.001	0.086	<0.001				
Objective support							−0.008	0.617	0.006	0.592
Subjective support							0.074	0.002	0.074	<0.001
Support utilization							0.065	0.001	0.011	0.279
**Depressive symptoms × Social support**					0.091	**0.043**				
Depressive symptoms × objective support									−0.062	0.014
Depressive symptoms × subjective support									0.044	0.374
Depressive symptoms × support utilization									0.213	**<0.001**
**Model fit**										
*F*	16.707 (< 0.001)		16.310 (< 0.001)		17.295(< 0.001)		69.289 (< 0.001)		15.068 (< 0.001)	
Adj *R^2^*	0.260		0.269		0.274		0.342		0.319	

ADL, Activities of daily living; IADL, Instrumental activities of daily living. Model 1–5 adjusted for age, gender, education, marital status, living arrangement, household income, smoking status, alcohol drinking status, chronic conditions, ADL, and IADL.

Based on model 2, model 4 included the three types of social support (objective support, subjective support, and support utilization) and the results indicated that subjective support and support utilization were significantly positively associated to cognitive function (*β* =0.074, *p* = 0.002; *β* =0.065, *p* = 0.001, respectively), and objective support was not associated with cognitive function. Model 5 included three interaction terms (depressive symptoms × objective support, depressive symptoms × subjective support, and depressive symptoms × support utilization, respectively) and the results showed that support utilization reduced the possibility of cognitive decline in older people with depressive symptoms (*β* = 0.213, *p* < 0.001), objective support and subjective support did not play a moderating role in the relationship between depressive symptoms and cognitive function. Figure 2B plots the estimated effects of support utilization on cognitive function by depressive symptoms. In the absence of support utilization, the cognitive score of older adults with depressive symptoms was 22.31, and that of without depressive symptoms older adults was 28.74. When support utilization was at the M + 1SD level, the cognitive score of older adults with depressive symptoms was 28.07, and that of without depressive symptoms older adults was 29.00.

**Figure 3:**
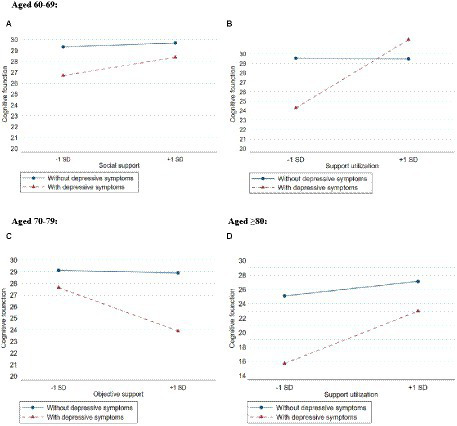
Moderation of social support (objective support, subjective support and support utilization) to depressive symptoms and cognitive function among different age group. **(A)** Represents depressive symptoms × social support in people aged 60–69 years. **(B)** Represents depressive symptoms × support utilization in people aged 60–69 years. **(C)** Represents depressive symptoms × objective support in people aged 70–79 years. **(D)** Represents depressive symptoms × support utilization in people aged ≥80 years.

For older adults aged 60–69, results showed that social support (depressive symptoms × social support) and support utilization (depressive symptoms × support utilization) reduced the possibility of cognitive decline in older people with depressive symptoms (*β* = 0.090, *p* = 0.003; *β* = 0.310, *p* < 0.001, respectively), objective support and subjective support did not play a moderating role in the relationship between depressive symptoms and cognitive function. The analysis of moderating effects is shown in Supplementary Table S6. Figure 3A plots the estimated effects of social support on cognitive function by depressive symptoms for older adults aged 60–69 years. In the absence of social support, the cognitive score of older adults with depressive symptoms was 26.70, and that of without depressive symptoms older adults was 29.34. When social support was at the M + 1SD level, the cognitive score of older adults with depressive symptoms was 28.37, and that of without depressive symptoms older adults was 29.70. Figure 3B plots the estimated effects of support utilization on cognitive function by depressive symptoms for older adults aged 60–69 years. In the absence of support utilization, the cognitive score of older adults with depressive symptoms was 24.29, and that of without depressive symptoms older adults was 29.54. When support utilization was at the M + 1SD level, the cognitive score of older adults with depressive symptoms was 31.51, and that of without depressive symptoms older adults was 29.48.

For older adults aged 70–79, the results showed that only objective support played a moderating role (depressive symptoms × objective support), which increased the possibility of cognitive decline in older people with depressive symptoms (*β* = −0.189, *p* < 0.001). The analysis of moderating effects is shown in Supplementary Table S7. Figure 3C plots the estimated effects of objective support on cognitive function by depressive symptoms for older adults aged 70–79 years. In the absence of objective support, the cognitive score of older adults with depressive symptoms was 27.63, and that of without depressive symptoms older adults was 29.11. When objective support was at the M + 1SD level, the cognitive score of older adults with depressive symptoms was 23.89, and that of without depressive symptoms older adults was 28.89.

For older adults aged 80 years and above, the results showed that only support utilization played a moderating role (depressive symptoms × support utilization), which reduced the possibility of cognitive decline in older people with depressive symptoms (*β* = 0.199, *p* < 0.001). The analysis of moderating effects is shown in Supplementary Table S8. Figure 3D plots the estimated effects of support utilization on cognitive function by depressive symptoms for older adults aged 80 years and above. In the absence of support utilization, the cognitive score of older adults with depressive symptoms was 15.68, and that of without depressive symptoms older adults was 25.10. When support utilization was at the M + 1SD level, the cognitive score of older adults with depressive symptoms was 22.97, and that of without depressive symptoms older adults was 27.13.

## 4. Discussion

This study aimed to explore the moderating effect of social support on the relationship between depressive symptoms and cognitive function. In addition to overall social support, our results highlight that the role played by different types of social support in the relationship between depressive symptoms and cognitive functioning varies and that there are certain differences in their influence across age groups. Specifically, overall social support and support utilization reduced the possibility of cognitive deterioration in depressed older adults, with support utilization having the strongest effect. We also found that objective support had the opposite effect. In addition, support utilization alleviated cognitive function in depressed older adults aged 60–69 years and ≥ 80 years, while objective support had a negative effect on cognitive functioning in depressed older adults aged 70–79 years.

Consistent with previous study, our findings supported the position that depressive symptoms are associated with cognitive function (Oh et al., 2021). Studies have shown that depression in older adults can lead to hippocampal atrophy (Ezzati et al., 2013), meningitis (Diniz et al., 2015), and amyloid deposition (Yasuno et al., 2016), all of which are pathological features of cognitive impairment. In addition, social contact can enhance cognitive reserve or encourage beneficial behaviors, whereas depression symptoms in older adults often manifest as alienation from friends and family and social avoidance, which is not conducive to promoting good cognitive function (Livingston et al., 2020).

This study found that total social support moderated the relationship between depressive symptoms and cognitive function. Specifically, support utilization reduced the possibility of cognitive deterioration in depressed older adults in this study. Compared with subjective support and objective support, support utilization emphasizes actively seeking help and making full use of the help sought, which assists older adults to acquire the social support they really require (Xiao, 1994). A study has shown that a higher degree of support utilization is conducive to social participation and that positive social interactions can reduce loneliness and social isolation of older adults, which has been shown to be an important influencing factor on cognition in older adults (Qian et al., 2017; Nie et al., 2021). Social interactions may be a positive influence on an individual’s lifestyle and health behaviors (e.g., exercising, smoking, going to the doctor, and adhering to medication regimens), thus contributing to better cognitive functioning (Seeman and Crimmins, 2001). Interestingly, this study found that support utilization was not only beneficial for alleviating decline in cognitive function in depressed older adults aged 60–69 years, but also in people aged 80 years and above. For those aged 60–69 years, a possible reason for this is that their physical health is still in a relatively good state and they thus have the ability to actively seek and make use of external resources. Moreover, as “survivors,” older adults aged 80 years and above have been shown to have a higher level of satisfaction, morale, and are more proactive in their lives. Therefore they may have a greater sense of initiative to seek and use outside help, although they are older and may have more physical limitations (Buono et al., 1998; von Heideken Wågert et al., 2005).

But for objective support, which increased the possibility of cognitive deterioration in depressed older adults in the age group of 70–79 years. Previous studies have shown that passively accepting objective support, especially unwanted instrumental support, may increase older adults’ feelings of helplessness and dependency and reduce their self-efficacy, which are risk factors for cognitive impairment (Uchino, 2009; Loomer et al., 2019). As of 2021, the average life expectancy of older adults in China is 78.2 years (National Health Commission of the People's Republic of China, 2022). For those who are aged 70–79, their health level starts to decline and some bothersome symptoms such as chronic diseases appear (Efraim and Jeremy, 2017). In this stage, they may receive more help and support from family, friends, and others, but cannot provide the same support in return. A study has shown that unequal exchange among social actors, such as over benefited, can lead to individual’s dissatisfaction and low mood, which lead to a decline of cognition in older adults (Stoller, 1985).

Moreover, the results of this study showed that subjective support did not have a moderating effect between depressive symptoms and cognitive function. Previous researches have shown that social support, especially emotional support, can increase self-esteem and engender a sense of belonging in older adults (Peggy, 2011; Ellwardt et al., 2013). Furthermore, it has been shown that social support in the form of supportive listening is associated with greater cognitive resilience and can independently modify the association between total brain volume and decreased cognitive function (Salinas et al., 2021). However, subjective support did not show the possibility of improving cognitive function in depressed older adults in this study, possibly because for depressed older adults, their cognitive function status is poor, and subjective support alone cannot improve their cognitive function level.

To the best of our knowledge, this is the first study to explore whether the moderating effect of different types of social support between depressive symptoms and cognitive function have different effects by age group, which provides a new perspective on reducing cognitive impairment in depressed older adults. Local governments and community committees should pay more attention to older adults with cognitive impairment who are depressed by providing them with the appropriate social support. Since support utilization, rather than objective support and subjective support, can alleviate the relationship between depressive symptoms and cognitive function, more attention should be paid to improving the ability of older adults to use social support. Meanwhile, targeted measures for older adults of different ages are also necessary. For those aged 60–69 years and 80 years and above, they should be educated in respect of effective ways to access outside help since they have the opportunity or are more willing to seek such assistance. As for those aged 70–79 years, it is important to avoid older adults producing a large psychological burden or leading them to becoming more dependent due to receiving too much objective support, and to consider encouraging them to take the initiative to take advantage of social support. In addition, encouraging older adults to participate in social activities may also be a measure to reduce cognitive decline. By participating in group activities, older adults can enrich their social support networks and promote the use of the social support around them.

Several limitations of this study should be considered when interpreting the findings. First, due to the cross-sectional design of this study, so this study is only an exploratory research, causal inferences cannot be made. We intend to conduct the first follow-up survey of the older adults surveyed in this study next year and will continue to do so at specific times in the future. Secondly, only the MMSE was used to measure cognitive function of older adults, which has a ceiling effect that may affect the measurement of cognitive function, and we will refine the tool for measuring cognitive function in future follow-up surveys. Third, only the GDS-30 was used to measure depressive symptoms, which may be less accurate than a formal rating from a clinical psychologist or psychiatrist. Finally, the sample selected from one city in China limits the generalizability of our findings.

## 5. Conclusion

In conclusion, this study indicates that the cognitive functioning in depressed older adults can be regulated by social support. Support utilization rather than subjective support and objective support reduced the possibility of cognitive deterioration in depressed older adults. The utilization of support in particular modified the association of depressive symptoms and cognitive function of aged 60–69 and 80 years and above, while for depressed older adults aged 70–79 years, we further found that too much objective support will increase the possibility of cognitive impairment in depressed older adults. Therefore, differences of age and different types of social support should be taken into account when providing depressed older adults with social support to attempt to improve their cognitive function.

## Data Availability Statement

The datasets presented in this article are not readily available because Data sharing is not applicable to this article due to privacy restrictions. Requests to access the datasets should be directed to corresponding author.

## Ethics Statement

The studies involving human participants were reviewed and approved by Ethical Review Board of Fudan University (NO. IRB#TYSQ 2019-2-03). The patients/participants provided their written informed consent to participate in this study.

## Author Contributions

YW: conception and design of the study, and critical revision of the manuscript. YR: critical revision of the manuscript. YJ: analysis of data and drafting of the manuscript. WW: drafting of the manuscript. WP: supervising the data analysis. MW, XC, XL, and PW: collection and curation of the data. FY, YY, XW, and SS participated in the research design. All authors contributed to the article and approved the submitted version.

## Funding

This work was supported by the National Key Research and Development Project (2017YFC1310504); the National Natural Science Foundation of China (71673055); and the Project of the Key Discipline Construction, Shanghai 3-Year Public Health Action Plan (GWV-10.1-XK18). The funder had no role in study design, data collection and analysis, decision to publish or preparation of the manuscript.

## Conflict of interest

The authors declare that the research was conducted in the absence of any commercial or financial relationships that could be construed as a potential conflict of interest.

## Publisher’s note

All claims expressed in this article are solely those of the authors and do not necessarily represent those of their affiliated organizations, or those of the publisher, the editors and the reviewers. Any product that may be evaluated in this article, or claim that may be made by its manufacturer, is not guaranteed or endorsed by the publisher.
