# Supplementary material for: Social support and cognitive function in Chinese older adults who experienced depressive symptoms: is there an age difference?
Source: Front Aging Neurosci. 2023 May 12;15:1175252. doi: 10.3389/fnagi.2023.1175252 (PMC10213363; doi:10.3389/fnagi.2023.1175252)
Supplement: Supplementary file 1 [file Table_1.DOCX]

Supplementary Material

**Social support and cognitive function in Chinese older adults who experienced depressive symptoms: Is there an age difference?**

**Yurong Jing^1, 2†,^ Wei Wang^1†^, Wenjia Peng^1, 2,^ Meng Wang^1, 2^, Xiaoli Chen^1, 2^, Xinya Liu^1, 2^, Pengfei Wang^1, 2^, Fei Yan^1^, Yinghua Yang^3^, Xinguo Wang^4^, Shuangyuan Sun^5^, Ye Ruan^5*^, Ying Wang^1, 2*^**

*** Correspondence:**

Prof. Ying Wang, PhD. School of Public Health, Fudan University, 130 DongAn Road, Shanghai 200032, China. E-mail address: wangying1013@fudan.edu.cn. Telephone: +86-21-33563936(FAX)

Ye Ruan, MD. Shanghai Municipal Center for Disease Control and Prevention, 1380 Zhongshan West Road, Shanghai 200336, China. E-mail address: ruanye@scdc.sh.cn

Prof. Ying Wang will handle correspondence at all stages of refereeing and publication, also post-publication.

# Supplementary Table

TABLE S1 Correlation matrix of variables.

| Variables | 1 | 2 | 3 | 4 | 5 | 6 | 7 | 8 | 9 | 10 | 11 | 12 | 13 | 14 | 15 | 16 | 17 |
| --- | --- | --- | --- | --- | --- | --- | --- | --- | --- | --- | --- | --- | --- | --- | --- | --- | --- |
| 1 Cognitive function | 1.000 |  |  |  |  |  |  |  |  |  |  |  |  |  |  |  |  |
| 2 Depressive symptoms | -0.308^*^ | 1.000 |  |  |  |  |  |  |  |  |  |  |  |  |  |  |  |
| 3 Social support | 0.289^*^ | -0.213^*^ | 1.000 |  |  |  |  |  |  |  |  |  |  |  |  |  |  |
| 4 Objective support | 0.098^*^ | -0.174^*^ | 0.365^*^ | 1.000 |  |  |  |  |  |  |  |  |  |  |  |  |  |
| 5 Subjective support | 0.301^*^ | -0.209^*^ | 0.879^*^ | 0.106^*^ | 1.000 |  |  |  |  |  |  |  |  |  |  |  |  |
| 6 Support utilization | 0.114^*^ | -0.012 | 0.586^*^ | -0.117^*^ | 0.357^*^ | 1.000 |  |  |  |  |  |  |  |  |  |  |  |
| 7 Age | -0.356^*^ | 0.243^*^ | -0.230^*^ | -0.118^*^ | -0.256^*^ | -0.026 | 1.000 |  |  |  |  |  |  |  |  |  |  |
| 8 Gender | -0.090^*^ | 0.052^*^ | -0.036 | -0.079^*^ | -0.041^*^ | 0.064^*^ | 0.028 | 1.000 |  |  |  |  |  |  |  |  |  |
| 9 Education | 0.259^*^ | -0.129^*^ | 0.155^*^ | 0.037 | 0.172^*^ | 0.053^*^ | -0.196^*^ | -0.158^*^ | 1.000 |  |  |  |  |  |  |  |  |
| 10 Marital status | 0.220^*^ | -0.158^*^ | 0.379^*^ | 0.397^*^ | 0.324^*^ | 0.039 | -0.282^*^ | -0.193^*^ | 0.189^*^ | 1.000 |  |  |  |  |  |  |  |
| 11 Living arrangement | -0.100^*^ | 0.088^*^ | -0.295^*^ | -0.338^*^ | -0.232^*^ | -0.026 | 0.117^*^ | 0.066^*^ | -0.068^*^ | -0.511^*^ | 1.000 |  |  |  |  |  |  |
| 12 Household income | 0.138^*^ | -0.159^*^ | 0.335^*^ | 0.334^*^ | 0.271^*^ | 0.086^*^ | -0.108^*^ | -0.099^*^ | 0.138^*^ | 0.330^*^ | -0.282^*^ | 1.000 |  |  |  |  |  |
| 13 Smoking status | 0.066^*^ | -0.047^*^ | 0.014 | -0.048^*^ | 0.039^*^ | -0.002 | -0.135^*^ | -0.409^*^ | 0.040^*^ | 0.055^*^ | -0.003 | 0.028 | 1.000 |  |  |  |  |
| 14 Alcohol drinking status | 0.021 | -0.062^*^ | 0.019 | -0.060^*^ | 0.048^*^ | 0.001 | -0.080^*^ | -0.340^*^ | 0.003 | 0.034 | -0.002 | 0.015 | 0.421^*^ | 1.000 |  |  |  |
| 15 Chronic conditions | -0.153^*^ | 0.207^*^ | -0.110^*^ | -0.131^*^ | -0.097^*^ | -0.004 | 0.260^*^ | 0.004 | -0.077^*^ | -0.100^*^ | 0.040^*^ | -0.064^*^ | -0.021 | 0.025 | 1.000 |  |  |
| 16 ADL | 0.289^*^ | -0.306^*^ | 0.187^*^ | 0.107^*^ | 0.199^*^ | 0.034 | -0.352^*^ | -0.074^*^ | 0.160^*^ | 0.154^*^ | -0.059^*^ | 0.113^*^ | 0.113^*^ | 0.052^*^ | -0.288^*^ | 1.000 |  |
| 17 IADL | 0.380^*^ | -0.445^*^ | 0.254^*^ | 0.201^*^ | 0.252^*^ | 0.022 | -0.406^*^ | -0.064^*^ | 0.184^*^ | 0.219^*^ | -0.087^*^ | 0.154^*^ | 0.058^*^ | 0.034 | -0.332^*^ | 0.565^*^ | 1.000 |

*p < 0.05.

TABLE S2 Correlation matrix of variables in the 60-69 age group.

| Variables | 1 | 2 | 3 | 4 | 5 | 6 | 7 | 8 | 9 | 10 | 11 | 12 | 13 | 14 | 15 | 16 |
| --- | --- | --- | --- | --- | --- | --- | --- | --- | --- | --- | --- | --- | --- | --- | --- | --- |
| 1 Cognitive function | 1.000 |  |  |  |  |  |  |  |  |  |  |  |  |  |  |  |
| 2 Depressive symptoms | -0.173^*^ | 1.000 |  |  |  |  |  |  |  |  |  |  |  |  |  |  |
| 3 Social support | 0.200^*^ | -0.160^*^ | 1.000 |  |  |  |  |  |  |  |  |  |  |  |  |  |
| 4 Objective support | 0.099^*^ | -0.142^*^ | 0.297^*^ | 1.000 |  |  |  |  |  |  |  |  |  |  |  |  |
| 5 Subjective support | 0.200^*^ | -0.130^*^ | 0.862^*^ | 0.027 | 1.000 |  |  |  |  |  |  |  |  |  |  |  |
| 6 Support utilization | 0.077^*^ | -0.044 | 0.638^*^ | -0.120^*^ | 0.381^*^ | 1.000 |  |  |  |  |  |  |  |  |  |  |
| 7 Gender | -0.043 | 0.048 | 0.047 | 0.006 | 0.026 | 0.070^*^ | 1.000 |  |  |  |  |  |  |  |  |  |
| 8 Education | 0.145^*^ | -0.106^*^ | 0.089^*^ | 0.018 | 0.086^*^ | 0.064^*^ | -0.077^*^ | 1.000 |  |  |  |  |  |  |  |  |
| 9 Marital status | 0.062^*^ | -0.103^*^ | 0.279^*^ | 0.303^*^ | 0.233^*^ | 0.041 | -0.064^*^ | 0.034 | 1.000 |  |  |  |  |  |  |  |
| 10 Living arrangement | -0.020 | 0.097^*^ | -0.245^*^ | -0.270^*^ | -0.197^*^ | -0.041 | 0.009 | -0.026 | -0.524^*^ | 1.000 |  |  |  |  |  |  |
| 11 Household income | 0.103^*^ | -0.157^*^ | 0.288^*^ | 0.329^*^ | 0.225^*^ | 0.070^*^ | -0.041 | 0.048 | 0.276^*^ | -0.243^*^ | 1.000 |  |  |  |  |  |
| 12 Smoking status | 0.001 | -0.022 | -0.050 | -0.101^*^ | -0.015 | -0.027 | -0.466^*^ | 0.013 | -0.030 | 0.033 | -0.007 | 1.000 |  |  |  |  |
| 13 Alcohol drinking status | -0.046 | -0.031 | -0.017 | -0.094^*^ | 0.017 | -0.015 | -0.372^*^ | -0.032 | -0.006 | 0.021 | 0.016 | 0.452^*^ | 1.000 |  |  |  |
| 14 Chronic conditions | -0.106^*^ | 0.130^*^ | -0.034 | -0.083^*^ | -0.028 | 0.013 | -0.011 | -0.088^*^ | -0.032 | -0.009 | -0.012 | 0.020 | 0.052^*^ | 1.000 |  |  |
| 15 ADL | 0.184^*^ | -0.237^*^ | 0.103^*^ | 0.035 | 0.114^*^ | 0.043 | -0.032 | 0.098^*^ | 0.005 | -0.008 | 0.069^*^ | 0.079^*^ | 0.021 | -0.224^*^ | 1.000 |  |
| 16 IADL | 0.258^*^ | -0.349^*^ | 0.178^*^ | 0.130^*^ | 0.157^*^ | 0.066^*^ | -0.028 | 0.143^*^ | 0.044 | -0.001 | 0.105^*^ | 0.016 | -0.017 | -0.271^*^ | 0.502^*^ | 1.000 |

*p < 0.05.

TABLE S3 Correlation matrix of variables in the 70-79 age group.

| Variables | 1 | 2 | 3 | 4 | 5 | 6 | 7 | 8 | 9 | 10 | 11 | 12 | 13 | 14 | 15 | 16 |
| --- | --- | --- | --- | --- | --- | --- | --- | --- | --- | --- | --- | --- | --- | --- | --- | --- |
| 1 Cognitive function | 1.000 |  |  |  |  |  |  |  |  |  |  |  |  |  |  |  |
| 2 Depressive symptoms | -0.270^*^ | 1.000 |  |  |  |  |  |  |  |  |  |  |  |  |  |  |
| 3 Social support | 0.166^*^ | -0.168^*^ | 1.000 |  |  |  |  |  |  |  |  |  |  |  |  |  |
| 4 Objective support | -0.109^*^ | -0.066 | 0.323^*^ | 1.000 |  |  |  |  |  |  |  |  |  |  |  |  |
| 5 Subjective support | 0.197^*^ | -0.185^*^ | 0.870^*^ | 0.041 | 1.000 |  |  |  |  |  |  |  |  |  |  |  |
| 6 Support utilization | 0.138^*^ | -0.012 | 0.599^*^ | -0.165^*^ | 0.381^*^ | 1.000 |  |  |  |  |  |  |  |  |  |  |
| 7 Gender | -0.088^*^ | 0.015 | -0.043 | -0.124^*^ | -0.032 | 0.067 | 1.000 |  |  |  |  |  |  |  |  |  |
| 8 Education | 0.182^*^ | -0.078^*^ | 0.054 | -0.050 | 0.087^*^ | 0.020 | -0.097^*^ | 1.000 |  |  |  |  |  |  |  |  |
| 9 Marital status | -0.017 | -0.036 | 0.302^*^ | 0.396^*^ | 0.233^*^ | -0.041 | -0.232^*^ | 0.018 | 1.000 |  |  |  |  |  |  |  |
| 10 Living arrangement | -0.083^*^ | 0.064 | -0.295^*^ | -0.361^*^ | -0.201^*^ | -0.011 | 0.110^*^ | -0.078^*^ | -0.590^*^ | 1.000 |  |  |  |  |  |  |
| 11 Household income | 0.047 | -0.086^*^ | 0.319^*^ | 0.269^*^ | 0.245^*^ | 0.112^*^ | -0.096^*^ | 0.136^*^ | 0.345^*^ | -0.303^*^ | 1.000 |  |  |  |  |  |
| 12 Smoking status | 0.052 | -0.028 | 0.022 | -0.015 | 0.019 | 0.029 | -0.352^*^ | -0.030 | 0.043 | -0.002 | 0.036 | 1.000 |  |  |  |  |
| 13 Alcohol drinking status | 0.016 | -0.054 | 0.014 | -0.048 | 0.031 | 0.023 | -0.306^*^ | -0.069 | 0.028 | -0.012 | -0.018 | 0.371^*^ | 1.000 |  |  |  |
| 14 Chronic conditions | 0.016 | 0.141^*^ | -0.110^*^ | -0.194^*^ | -0.066 | -0.003 | 0.022 | 0.007 | -0.070 | 0.066 | -0.057 | -0.012 | 0.048 | 1.000 |  |  |
| 15 ADL | 0.124^*^ | -0.253^*^ | 0.102^*^ | 0.090^*^ | 0.090^*^ | 0.015 | -0.103^*^ | 0.030 | 0.078^*^ | -0.093^*^ | 0.099^*^ | 0.057 | 0.038 | -0.219^*^ | 1.000 |  |
| 16 IADL | 0.190^*^ | -0.420^*^ | 0.118^*^ | 0.138^*^ | 0.120^*^ | -0.030 | -0.045 | 0.031 | 0.063 | -0.104^*^ | 0.111^*^ | -0.014 | 0.036 | -0.310^*^ | 0.490^*^ | 1.000 |

*p < 0.05.

TABLE S4 Correlation matrix of variables in the 80 years and above age group.

| Variables | 1 | 2 | 3 | 4 | 5 | 6 | 7 | 8 | 9 | 10 | 11 | 12 | 13 | 14 | 15 | 16 |
| --- | --- | --- | --- | --- | --- | --- | --- | --- | --- | --- | --- | --- | --- | --- | --- | --- |
| 1 Cognitive function | 1.000 |  |  |  |  |  |  |  |  |  |  |  |  |  |  |  |
| 2 Depressive symptoms | -0.339^*^ | 1.000 |  |  |  |  |  |  |  |  |  |  |  |  |  |  |
| 3 Social support | 0.348^*^ | -0.152^*^ | 1.000 |  |  |  |  |  |  |  |  |  |  |  |  |  |
| 4 Objective support | 0.124^*^ | -0.257^*^ | 0.554^*^ | 1.000 |  |  |  |  |  |  |  |  |  |  |  |  |
| 5 Subjective support | 0.366^*^ | -0.147^*^ | 0.891^*^ | 0.288^*^ | 1.000 |  |  |  |  |  |  |  |  |  |  |  |
| 6 Support utilization | 0.210^*^ | 0.110^*^ | 0.455^*^ | -0.057 | 0.264^*^ | 1.000 |  |  |  |  |  |  |  |  |  |  |
| 7 Gender | -0.220^*^ | 0.074 | -0.293^*^ | -0.289^*^ | -0.283^*^ | 0.050 | 1.000 |  |  |  |  |  |  |  |  |  |
| 8 Education | 0.315^*^ | -0.023 | 0.223^*^ | 0.058 | 0.274^*^ | 0.032 | -0.461^*^ | 1.000 |  |  |  |  |  |  |  |  |
| 9 Marital status | 0.304^*^ | -0.073 | 0.512^*^ | 0.529^*^ | 0.401^*^ | 0.110^*^ | -0.436^*^ | 0.340^*^ | 1.000 |  |  |  |  |  |  |  |
| 10 Living arrangement | -0.018 | -0.008 | -0.346^*^ | -0.472^*^ | -0.245^*^ | 0.003 | 0.122^*^ | -0.008 | -0.389^*^ | 1.000 |  |  |  |  |  |  |
| 11 Household income | 0.194^*^ | -0.167^*^ | 0.406^*^ | 0.417^*^ | 0.320^*^ | 0.077 | -0.301^*^ | 0.248^*^ | 0.406^*^ | -0.316^*^ | 1.000 |  |  |  |  |  |
| 12 Smoking status | 0.026 | 0.046 | 0.105^*^ | 0.030 | 0.111^*^ | 0.053 | -0.196^*^ | 0.103 | 0.133^*^ | -0.023 | 0.094 | 1.000 |  |  |  |  |
| 13 Alcohol drinking status | 0.055 | -0.072 | 0.062 | -0.006 | 0.091 | 0.018 | -0.231^*^ | 0.159^*^ | 0.016 | 0.008 | 0.008 | 0.174^*^ | 1.000 |  |  |  |
| 14 Chronic conditions | -0.146^*^ | 0.308^*^ | -0.082 | -0.077 | -0.046 | -0.050 | 0.006 | 0.060 | -0.037 | 0.015 | -0.124^*^ | 0.033 | 0.009 | 1.000 |  |  |
| 15 ADL | 0.260^*^ | -0.262^*^ | 0.124^*^ | 0.110^*^ | 0.121^*^ | -0.046 | -0.127^*^ | 0.142^*^ | 0.072 | 0.110^*^ | 0.089 | 0.022 | -0.016 | -0.242^*^ | 1.000 |  |
| 16 IADL | 0.351^*^ | -0.395^*^ | 0.194^*^ | 0.249^*^ | 0.182^*^ | -0.096 | -0.128^*^ | 0.084 | 0.160^*^ | 0.023 | 0.176^*^ | -0.060 | -0.038 | -0.224^*^ | 0.454^*^ | 1.000 |

*p < 0.05.

TABLE S5 Moderating effects of social support (objective support, subjective support, and support utilization) on the relationship between depressive symptoms and cognitive function.

|  | **Model 1** | |  | **Model 2** | |  | **Model 3** | |  | **Model 4** | |  | **Model 5** | |
| --- | --- | --- | --- | --- | --- | --- | --- | --- | --- | --- | --- | --- | --- | --- |
|  | ***β*** | ***p*-value** |  | ***β*** | ***p*-value** |  | ***β*** | ***p-*value** |  | ***β*** | ***p*-value** |  | β | ***p*-value** |
| **Depressive symptoms (ref: no)** |  |  |  |  | |  |  | |  |  | |  |  | |
| Yes | -0.286 | <0.001 |  | -0.277 | <0.001 |  | -0.241 | <0.001 |  | -0.279 | <0.001 |  | -0.252 | <0.001 |
| **Social support** |  |  |  | 0.108 | <0.001 |  | 0.086 | <0.001 |  |  |  |  |  |  |
| [Objective support](javascript:;) |  |  |  |  |  |  |  |  |  | -0.008 | 0.617 |  | 0.006 | 0.592 |
| [Subjective support](javascript:;) |  |  |  |  |  |  |  |  |  | 0.074 | 0.002 |  | 0.074 | <0.001 |
| Support utilization |  |  |  |  |  |  |  |  |  | 0.065 | 0.001 |  | 0.011 | 0.279 |
| **Depressive symptoms × Social support** |  |  |  |  |  |  | 0.091 | **0.043** |  |  |  |  |  |  |
| Depressive symptoms × [objective support](javascript:;) |  |  |  |  |  |  |  |  |  |  |  |  | -0.062 | 0.014 |
| Depressive symptoms × [subjective support](javascript:;) |  |  |  |  |  |  |  |  |  |  |  |  | 0.044 | 0.374 |
| Depressive symptoms × support utilization |  |  |  |  |  |  |  |  |  |  |  |  | 0.213 | **<0.001** |
| **Age (ref: 60 – 69)** |  |  |  |  |  |  |  |  |  |  |  |  |  |  |
| 70 – 79 | -0.015 | 0.405 |  | -0.011 | 0.550 |  | -0.014 | 0.419 |  | -0.010 | 0.592 |  | -0.012 | 0.495 |
| ≥ 80 | -0.168 | <0.001 |  | -0.160 | <0.001 |  | -0.162 | <0.001 |  | -0.160 | <0.001 |  | -0.176 | <0.001 |
| **Gender (ref: male)** |  |  |  |  |  |  |  |  |  |  |  |  |  |  |
| Female | -0.003 | 0.871 |  | -0.009 | 0.624 |  | -0.011 | 0.571 |  | -0.017 | 0.370 |  | -0.025 | 0.176 |
| **Education (ref: Illiterate / Primary school)** |  |  |  |  |  |  |  |  |  |  |  |  |  |  |
| Junior school | 0.211 | <0.001 |  | 0.206 | <0.001 |  | 0.203 | <0.001 |  | 0.202 | <0.001 |  | 0.200 | <0.001 |
| Senior high school | 0.250 | <0.001 |  | 0.242 | <0.001 |  | 0.243 | <0.001 |  | 0.235 | <0.001 |  | 0.229 | <0.001 |
| College or above | 0.208 | <0.001 |  | 0.201 | <0.001 |  | 0.200 | <0.001 |  | 0.194 | <0.001 |  | 0.193 | <0.001 |
| **Marital status (ref: Unmarried/divorced/widowed)** | | |  |  |  |  |  |  |  |  |  |  |  |  |
| Married | 0.102 | <0.001 |  | 0.080 | <0.001 |  | 0.078 | 0.001 |  | 0.093 | <0.001 |  | 0.078 | <0.001 |
| **Living arrangement (ref: with others)** |  |  |  |  |  |  |  |  |  |  |  |  |  |  |
| Alone | 0.082 | <0.001 |  | 0.099 | <0.001 |  | 0.102 | <0.001 |  | 0.082 | <0.001 |  | 0.068 | <0.001 |
| **Household income (ref:** ≤ **5,000)** |  |  |  |  |  |  |  |  |  |  |  |  |  |  |
| 5,001-10,000 | 0.052 | 0.089 |  | 0.028 | 0.369 |  | 0.021 | 0.502 |  | 0.038 | 0.215 |  | 0.046 | 0.121 |
| > 10,000 | 0.031 | 0.301 |  | -0.009 | 0.769 |  | -0.015 | 0.615 |  | 0.012 | 0.701 |  | 0.015 | 0.606 |
| **Smoking status (ref: Never/Past)** |  |  |  |  |  |  |  |  |  |  |  |  |  |  |
| Current | 0.001 | 0.963 |  | 0.002 | 0.902 |  | 0.003 | 0.879 |  | -0.003 | 0.885 |  | -0.004 | 0.825 |
| **Alcohol drinking status (ref: Never/Past)** |  |  |  |  |  |  |  |  |  |  |  |  |  |  |
| Current | 0.005 | 0.799 |  | 0.004 | 0.841 |  | 0.003 | 0.877 |  | -0.001 | 0.945 |  | -0.007 | 0.714 |
| **Chronic conditions (ref: No)** |  |  |  |  |  |  |  |  |  |  |  |  |  |  |
| Yes | 0.009 | 0.635 |  | 0.010 | 0.562 |  | 0.009 | 0.625 |  | 0.005 | 0.787 |  | 0.007 | 0.700 |
| **ADL** (**ref:** **impaired**) |  |  |  |  |  |  |  |  |  |  |  |  |  |  |
| Normal | 0.024 | 0.251 |  | 0.023 | 0.248 |  | 0.023 | 0.249 |  | 0.014 | 0.502 |  | 0.016 | 0.416 |
| **IADL (ref: impaired)** |  |  |  |  |  |  |  |  |  |  |  |  |  |  |
| Normal | 0.115 | <0.001 |  | 0.104 | <0.001 |  | 0.104 | <0.001 |  | 0.114 | <0.001 |  | 0.113 | <0.001 |
| **Model fit** |  |  |  |  |  |  |  |  |  |  |  |  |  |  |
| *F* | 16.707 (< 0.001) | |  | 16.310 (< 0.001) | |  | 17.295(< 0.001) | |  | 69.289 (< 0.001) | |  | 15.068 (< 0.001) | |
| Adj *R^2^* | 0.260 | |  | 0.269 | |  | 0.274 | |  | 0.342 | |  | 0.319 | |

ADL, Activities of daily living; IADL, Instrumental activities of daily living. Model 1-5 adjusted for age, gender, education, marital status, living arrangement, household income, smoking status, alcohol drinking status, chronic conditions, ADL, and IADL.

TABLE S6 Moderating effects of social support (objective support, subjective support, and support utilization) on the relationship between depressive symptoms and cognitive function in the 60-69 age group.

|  | **Model 1** | |  | **Model 2** | |  | **Model 3** | |  | **Model 4** | |  | **Model 5** | |
| --- | --- | --- | --- | --- | --- | --- | --- | --- | --- | --- | --- | --- | --- | --- |
|  | ***β*** | ***p*-value** |  | ***β*** | ***p*-value** |  | ***β*** | ***p-*value** |  | ***β*** | ***p*-value** |  | β | ***p*-value** |
| **Depressive symptoms (ref: no)** |  |  |  |  | |  |  | |  |  | |  |  | |
| Yes | -0.248 | <0.001 |  | -0.241 | <0.001 |  | -0.197 | <0.001 |  | -0.242 | <0.001 |  | -0.161 | <0.001 |
| **Social support** |  |  |  | 0.094 | 0.001 |  | 0.078 | 0.005 |  |  |  |  |  |  |
| [Objective support](javascript:;) |  |  |  |  |  |  |  |  |  | 0.042 | 0.130 |  | 0.033 | 0.213 |
| [Subjective support](javascript:;) |  |  |  |  |  |  |  |  |  | 0.045 | 0.108 |  | 0.081 | 0.004 |
| Support utilization |  |  |  |  |  |  |  |  |  | 0.056 | 0.033 |  | -0.014 | 0.580 |
| **Depressive symptoms × Social support** |  |  |  |  |  |  | 0.090 | **0.003** |  |  |  |  |  |  |
| Depressive symptoms × [objective support](javascript:;) |  |  |  |  |  |  |  |  |  |  |  |  | -0.006 | 0.844 |
| Depressive symptoms × [subjective support](javascript:;) |  |  |  |  |  |  |  |  |  |  |  |  | 0.006 | 0.849 |
| Depressive symptoms × support utilization |  |  |  |  |  |  |  |  |  |  |  |  | 0.310 | **<0.001** |
| **Gender (ref: male)** |  |  |  |  |  |  |  |  |  |  |  |  |  |  |
| Female | 0.033 | 0.248 |  | 0.026 | 0.358 |  | 0.023 | 0.417 |  | 0.026 | 0.360 |  | 0.022 | 0.412 |
| **Education (ref: Illiterate / Primary school)** |  |  |  |  |  |  |  |  |  |  |  |  |  |  |
| Junior school | 0.136 | 0.070 |  | 0.134 | 0.072 |  | 0.126 | 0.091 |  | 0.135 | 0.071 |  | 0.140 | 0.048 |
| Senior high school | 0.188 | 0.014 |  | 0.183 | 0.017 |  | 0.182 | 0.017 |  | 0.183 | 0.017 |  | 0.193 | 0.008 |
| College or above | 0.142 | 0.006 |  | 0.137 | 0.007 |  | 0.134 | 0.008 |  | 0.137 | 0.007 |  | 0.152 | 0.002 |
| **Marital status (ref: Unmarried/divorced/widowed)** | | |  |  |  |  |  |  |  |  |  |  |  |  |
| Married | 0.009 | 0.769 |  | -0.003 | 0.913 |  | -0.001 | 0.985 |  | -0.002 | 0.945 |  | -0.028 | 0.321 |
| **Living arrangement (ref: with others)** |  |  |  |  |  |  |  |  |  |  |  |  |  |  |
| Alone | 0.064 | 0.030 |  | 0.076 | 0.010 |  | 0.084 | 0.005 |  | 0.075 | 0.011 |  | 0.062 | 0.029 |
| **Household income (ref:** ≤ **5,000)** |  |  |  |  |  |  |  |  |  |  |  |  |  |  |
| 5,001-10,000 | 0.144 | 0.004 |  | 0.116 | 0.021 |  | 0.103 | 0.041 |  | 0.118 | 0.019 |  | 0.143 | 0.003 |
| > 10,000 | 0.139 | 0.005 |  | 0.099 | 0.053 |  | 0.086 | 0.090 |  | 0.101 | 0.050 |  | 0.119 | 0.015 |
| **Smoking status (ref: Never/Past)** |  |  |  |  |  |  |  |  |  |  |  |  |  |  |
| Current | -0.012 | 0.690 |  | -0.009 | 0.758 |  | -0.007 | 0.790 |  | -0.009 | 0.761 |  | -0.004 | 0.887 |
| **Alcohol drinking status (ref: Never/Past)** |  |  |  |  |  |  |  |  |  |  |  |  |  |  |
| Current | 0.017 | 0.544 |  | 0.016 | 0.575 |  | 0.016 | 0.569 |  | 0.017 | 0.551 |  | 0.012 | 0.638 |
| **Chronic conditions (ref: No)** |  |  |  |  |  |  |  |  |  |  |  |  |  |  |
| Yes | -0.014 | 0.575 |  | -0.015 | 0.558 |  | -0.019 | 0.463 |  | -0.015 | 0.560 |  | -0.013 | 0.594 |
| **ADL** (**ref:** **impaired**) |  |  |  |  |  |  |  |  |  |  |  |  |  |  |
| Normal | 0.049 | 0.081 |  | 0.050 | 0.075 |  | 0.049 | 0.083 |  | 0.051 | 0.075 |  | 0.051 | 0.062 |
| **IADL (ref: impaired)** |  |  |  |  |  |  |  |  |  |  |  |  |  |  |
| Normal | 0.131 | <0.001 |  | 0.119 | <0.001 |  | 0.111 | <0.001 |  | 0.119 | <0.001 |  | 0.112 | <0.001 |
| **Model fit** |  |  |  |  |  |  |  |  |  |  |  |  |  |  |
| *F* | 18.241 (<0.001) | |  | 17.937 (<0.001) | |  | 17.442 (<0.001) | |  | 15.855 (<0.001) | |  | 23.005 (<0.001) | |
| Adj *R^2^* | 0.141 | |  | 0.147 | |  | 0.152 | |  | 0.146 | |  | 0.230 | |

ADL, Activities of daily living; IADL, Instrumental activities of daily living. Model 1-5 adjusted for gender, education, marital status, living arrangement, household income, smoking status, alcohol drinking status, chronic conditions, ADL, and IADL.

TABLE S7 Moderating effects of social support (objective support, subjective support, and support utilization) on the relationship between depressive symptoms and cognitive function in the 70-79 age group.

|  | **Model 1** | |  | **Model 2** | |  | **Model 3** | |  | **Model 4** | |  | **Model 5** | |
| --- | --- | --- | --- | --- | --- | --- | --- | --- | --- | --- | --- | --- | --- | --- |
|  | ***β*** | ***p*-value** |  | ***β*** | ***p*-value** |  | ***β*** | ***p-*value** |  | ***β*** | ***p*-value** |  | β | ***p*-value** |
| **Depressive symptoms (ref: no)** |  |  |  |  | |  |  | |  |  | |  |  | |
| Yes | -0.313 | <0.001 |  | -0.302 | <0.001 |  | -0.309 | <0.001 |  | -0.297 | <0.001 |  | -0.296 | <0.001 |
| **Social support** |  |  |  | 0.096 | 0.015 |  | 0.105 | 0.014 |  |  |  |  |  |  |
| [Objective support](javascript:;) |  |  |  |  |  |  |  |  |  | -0.096 | 0.019 |  | -0.030 | 0.483 |
| [Subjective support](javascript:;) |  |  |  |  |  |  |  |  |  | 0.079 | 0.051 |  | 0.061 | 0.163 |
| Support utilization |  |  |  |  |  |  |  |  |  | 0.083 | 0.031 |  | 0.058 | 0.145 |
| **Depressive symptoms × Social support** |  |  |  |  |  |  | -0.023 | 0.576 |  |  |  |  |  |  |
| Depressive symptoms × [objective support](javascript:;) |  |  |  |  |  |  |  |  |  |  |  |  | -0.189 | **<0.001** |
| Depressive symptoms × [subjective support](javascript:;) |  |  |  |  |  |  |  |  |  |  |  |  | 0.051 | 0.273 |
| Depressive symptoms × support utilization |  |  |  |  |  |  |  |  |  |  |  |  | 0.066 | 0.106 |
| **Gender (ref: male)** |  |  |  |  |  |  |  |  |  |  |  |  |  |  |
| Female | -0.029 | 0.461 |  | -0.033 | 0.410 |  | -0.032 | 0.415 |  | -0.046 | 0.245 |  | -0.059 | 0.128 |
| **Education (ref: Illiterate / Primary school)** |  |  |  |  |  |  |  |  |  |  |  |  |  |  |
| Junior school | 0.238 | <0.001 |  | 0.225 | 0.001 |  | 0.224 | 0.001 |  | 0.222 | 0.001 |  | 0.201 | 0.002 |
| Senior high school | 0.259 | <0.001 |  | 0.249 | <0.001 |  | 0.248 | <0.001 |  | 0.238 | <0.001 |  | 0.223 | <0.001 |
| College or above | 0.207 | <0.001 |  | 0.201 | 0.001 |  | 0.200 | 0.001 |  | 0.187 | 0.001 |  | 0.178 | 0.002 |
|  | | |  |  |  |  |  |  |  |  |  |  |  |  |
| Married | -0.078 | 0.105 |  | -0.090 | 0.060 |  | -0.090 | 0.060 |  | -0.069 | 0.154 |  | -0.063 | 0.185 |
| **Living arrangement (ref: with others)** |  |  |  |  |  |  |  |  |  |  |  |  |  |  |
| Alone | -0.058 | 0.198 |  | -0.042 | 0.348 |  | -0.043 | 0.344 |  | -0.075 | 0.101 |  | -0.082 | 0.065 |
| **Household income (ref:** ≤ **5,000)** |  |  |  |  |  |  |  |  |  |  |  |  |  |  |
| 5,001-10,000 | -0.055 | 0.414 |  | -0.072 | 0.286 |  | -0.073 | 0.283 |  | -0.052 | 0.436 |  | -0.042 | 0.527 |
| > 10,000 | -0.076 | 0.246 |  | -0.109 | 0.102 |  | -0.110 | 0.102 |  | -0.080 | 0.228 |  | -0.069 | 0.295 |
| **Smoking status (ref: Never/Past)** |  |  |  |  |  |  |  |  |  |  |  |  |  |  |
| Current | 0.039 | 0.319 |  | 0.038 | 0.330 |  | 0.038 | 0.327 |  | 0.033 | 0.397 |  | 0.020 | 0.592 |
| **Alcohol drinking status (ref: Never/Past)** |  |  |  |  |  |  |  |  |  |  |  |  |  |  |
| Current | 0.020 | 0.613 |  | 0.020 | 0.611 |  | 0.020 | 0.605 |  | 0.008 | 0.845 |  | 0.010 | 0.784 |
| **Chronic conditions (ref: No)** |  |  |  |  |  |  |  |  |  |  |  |  |  |  |
| Yes | 0.046 | 0.215 |  | 0.054 | 0.147 |  | 0.055 | 0.141 |  | 0.034 | 0.359 |  | 0.041 | 0.267 |
| **ADL** (**ref:** **impaired**) |  |  |  |  |  |  |  |  |  |  |  |  |  |  |
| Normal | 0.026 | 0.523 |  | 0.026 | 0.519 |  | 0.026 | 0.524 |  | 0.016 | 0.697 |  | 0.010 | 0.798 |
| **IADL (ref: impaired)** |  |  |  |  |  |  |  |  |  |  |  |  |  |  |
| Normal | 0.141 | 0.001 |  | 0.141 | 0.001 |  | 0.140 | 0.002 |  | 0.150 | 0.001 |  | 0.152 | <0.001 |
| **Model fit** |  |  |  |  |  |  |  |  |  |  |  |  |  |  |
| *F* | 11.580 (<0.001) | |  | 11.288 (<0.001) | |  | 10.591 (<0.001) | |  | 11.056 (<0.001) | |  | 11.455 (<0.001) | |
| Adj *R^2^* | 0.181 | |  | 0.206 | |  | 0.187 | |  | 0.204 | |  | 0.238 | |

ADL, Activities of daily living; IADL, Instrumental activities of daily living. Model 1-5 adjusted for gender, education, marital status, living arrangement, household income, smoking status, alcohol drinking status, chronic conditions, ADL, and IADL.

TABLE S8 Moderating effects of social support (objective support, subjective support, and support utilization) on the relationship between depressive symptoms and cognitive function in the 80 years and above age group.

|  | **Model 1** | |  | **Model 2** | |  | **Model 3** | |  | **Model 4** | |  | **Model 5** | |
| --- | --- | --- | --- | --- | --- | --- | --- | --- | --- | --- | --- | --- | --- | --- |
|  | ***β*** | ***p*-value** |  | ***β*** | ***p*-value** |  | ***β*** | ***p-*value** |  | ***β*** | ***p*-value** |  | β | ***p*-value** |
| **Depressive symptoms (ref: no)** |  |  |  |  | |  |  | |  |  | |  |  | |
| Yes | -0.355 | <0.001 |  | -0.337 | <0.001 |  | -0.329 | <0.001 |  | -0.377 | <0.001 |  | -0.399 | <0.001 |
| **Social support** |  |  |  | 0.240 | <0.001 |  | 0.211 | 0.001 |  |  |  |  |  |  |
| [Objective support](javascript:;) |  |  |  |  |  |  |  |  |  | -0.213 | 0.026 |  | -0.066 | 0.287 |
| [Subjective support](javascript:;) |  |  |  |  |  |  |  |  |  | 0.130 | 0.010 |  | 0.143 | 0.017 |
| Support utilization |  |  |  |  |  |  |  |  |  | 0.253 | <0.001 |  | 0.130 | 0.019 |
| **Depressive symptoms × Social support** |  |  |  |  |  |  | 0.055 | 0.300 |  |  |  |  |  |  |
| Depressive symptoms × [objective support](javascript:;) |  |  |  |  |  |  |  |  |  |  |  |  | -0.074 | 0.165 |
| Depressive symptoms × [subjective support](javascript:;) |  |  |  |  |  |  |  |  |  |  |  |  | -0.009 | 0.867 |
| Depressive symptoms × support utilization |  |  |  |  |  |  |  |  |  |  |  |  | 0.199 | **<0.001** |
| **Gender (ref: male)** |  |  |  |  |  |  |  |  |  |  |  |  |  |  |
| Female | 0.009 | 0.871 |  | 0.012 | 0.822 |  | 0.009 | 0.871 |  | -0.035 | 0.508 |  | -0.031 | 0.548 |
| **Education (ref: Illiterate / Primary school)** |  |  |  |  |  |  |  |  |  |  |  |  |  |  |
| Junior school | 0.029 | 0.562 |  | 0.035 | 0.470 |  | 0.037 | 0.441 |  | 0.030 | 0.509 |  | 0.044 | 0.336 |
| Senior high school | 0.124 | 0.018 |  | 0.128 | 0.012 |  | 0.127 | 0.013 |  | 0.111 | 0.024 |  | 0.100 | 0.038 |
| College or above | 0.191 | <0.001 |  | 0.179 | 0.001 |  | 0.178 | 0.001 |  | 0.130 | 0.012 |  | 0.145 | 0.005 |
|  | | |  |  |  |  |  |  |  |  |  |  |  |  |
| Married | 0.219 | <0.001 |  | 0.135 | 0.026 |  | 0.134 | 0.028 |  | 0.160 | 0.006 |  | 0.134 | 0.021 |
| **Living arrangement (ref: with others)** |  |  |  |  |  |  |  |  |  |  |  |  |  |  |
| Alone | 0.102 | 0.046 |  | 0.144 | 0.005 |  | 0.144 | 0.005 |  | 0.065 | 0.199 |  | 0.057 | 0.247 |
| **Household income (ref:** ≤ **5,000)** |  |  |  |  |  |  |  |  |  |  |  |  |  |  |
| 5,001-10,000 | 0.043 | 0.506 |  | 0.029 | 0.639 |  | 0.026 | 0.675 |  | 0.077 | 0.203 |  | 0.075 | 0.211 |
| > 10,000 | -0.007 | 0.919 |  | -0.054 | 0.402 |  | -0.055 | 0.388 |  | -0.007 | 0.907 |  | 0.001 | 0.987 |
| **Smoking status (ref: Never/Past)** |  |  |  |  |  |  |  |  |  |  |  |  |  |  |
| Current | 0.007 | 0.873 |  | -0.001 | 0.993 |  | 0.001 | 0.979 |  | -0.018 | 0.685 |  | 0.006 | 0.890 |
| **Alcohol drinking status (ref: Never/Past)** |  |  |  |  |  |  |  |  |  |  |  |  |  |  |
| Current | -0.018 | 0.702 |  | -0.028 | 0.548 |  | -0.030 | 0.514 |  | -0.040 | 0.368 |  | -0.051 | 0.240 |
| **Chronic conditions (ref: No)** |  |  |  |  |  |  |  |  |  |  |  |  |  |  |
| Yes | -0.007 | 0.886 |  | -0.004 | 0.932 |  | -0.003 | 0.948 |  | 0.016 | 0.730 |  | 0.014 | 0.761 |
| **ADL** (**ref:** **impaired**) |  |  |  |  |  |  |  |  |  |  |  |  |  |  |
| Normal | 0.027 | 0.614 |  | 0.029 | 0.573 |  | 0.029 | 0.573 |  | 0.028 | 0.574 |  | 0.028 | 0.559 |
| **IADL (ref: impaired)** |  |  |  |  |  |  |  |  |  |  |  |  |  |  |
| Normal | 0.108 | 0.048 |  | 0.084 | 0.117 |  | 0.088 | 0.100 |  | 0.138 | 0.008 |  | 0.121 | 0.019 |
| **Model fit** |  |  |  |  |  |  |  |  |  |  |  |  |  |  |
| *F* | 11.294 (<0.001) | |  | 12.434 (<0.001) | |  | 11.727 (<0.001) | |  | 14.092 (<0.001) | |  | 13.316 (<0.001) | |
| Adj *R^2^* | 0.288 | |  | 0.325 | |  | 0.325 | |  | 0.385 | |  | 0.409 | |

ADL, Activities of daily living; IADL, Instrumental activities of daily living. Model 1-5 adjusted for gender, education, marital status, living arrangement, household income, smoking status, alcohol drinking status, chronic conditions, ADL, and IADL.
